# The Essential Oils and Some Other Agents—Their Antiseptic Value; Also, Their Irritating or Non Irritating Properties.—Abstracts

**Published:** 1898-08

**Authors:** A. H. Peck

**Affiliations:** Chicago, Ills.


THE DENTAL REGISTER.

VOL. LII.] AUGUST, 1898. [No. 8.

### COMMUNICATIONS.

### The Essential Oils and Some Other Agents—Their Antiseptic Value; also, Their Irritating or Non-Irritating Properties.—Abstracts.

BY A. H. PECK, M.D., D.D.S., CHICAGO, ILLS.

Read before the Dental Section of the American Medical Association, June, 1898.

Soon after assuming the duties of the Chair of Materia Medica and Therapeutics in college work I became convinced that in our literature there was much loose statement in regard to the action of the drugs we employ in dentistry. Especially did this seem true in regard to the antiseptic powers of the various agents employed as antiseptics. It seems that iodoform is still used by many as an anti-septic, though it has long been known that it has not that power. Also, that the presence of albumin renders the ordinary solutions of bichloride of mercury inert as to antiseptic power, and prevents its effectiveness in treating suppurative surfaces, yet it is persistently used for this purpose. Also, the essential oils, some of which have previously been shown to possess antiseptic virtues, have seemed to be looked upon as a group of antiseptics, and, as it has seemed to me, are being used without reference to their relative merits as antiseptics, or to their therapeutic effects upon the tissues to which they are applied. For these reasons I have, in my teaching in the Northwestern University Dental School, made trial of these agents in the bacteriological laboratory, as to their effectiveness as antiseptics, and have also in various ways made trial of their effects upon the animal tissues in order that I might speak definitely of my own knowledge of these matters. In this paper I will give briefly my observations upon a number of these agents.

To determine the antiseptic value of these agents the following experiments were conducted: Test tubes, each containing ten c. c. of sterilized mutton bouillon (which amount will hereafter be referred to as the *unit of culture media*) were used. The broth in these tubes were, for the most part, infected with saliva from various members of the class. In each set of plants made a control tube was used, *i. e.*, a tube in which the broth was infected with saliva, but into which no antiseptic agent was placed, simply to act as a control for the results of the remaining tubes into which antiseptic agents were placed. In each instance the control tube presented a full development of bacteria, thus proving the accuracy of each set of plants. One drop of the essential oil was first used in the tubes. When one drop prevented development of bacteria, the quantity was gradually decreased in other plants, until the least amount that would prevent development was ascertained. To divide the drop I placed ten drops of alcohol in a small vial, and into this placed one drop of the oil; the alcohol dissolves the oil immediately. I then used in the culture tube such proportion of the drop of the essential oil desired, one drop of the solution representing one-tenth of the drop of oil. Those drugs that were found ineffective with one drop were increased in other plants until found effective, or were given up as unsuitable or worthless as antiseptics. The same dropper was used throughout. When using the same dropper it will be observed that a drop of alcohol is smaller in bulk than a drop of an essential oil. Because of this difference in the size of the drops, ten drops of alcohol and one drop of an essential oil forms as nearly as it can be figured a ten percent solution. And one drop of this solution represents one-tenth of the drop of oil.

An antiseptic must be regarded as a poison to the vegetable cell, and many of them act also as poisons to the animal cell. I undertook this series of experiments for the determination of these differences of poisonous effects with the idea that in selecting antiseptics for use in practice we should have special regard to the effect of the agents upon the animal tissue to which they are applied. To determine the irritating or non-irritating properties of these oils an extensive course of experiments with them has been conducted during the winter months, in connection with sores artificially produced on guinea pigs, and also on my own person. To determine the effect of these agents when applied directly to soft tissue, the applications were made, in each instance, to my own person. And pardon me when I say that I believe I have come to positive conclusions regarding some of the agents along these lines.

Oil of Cassia.—We find that $\frac{3}{10}$ of a drop is the smallest quantity that will prevent the development of bacteria in the *unit of culture media* and there being sixty-seven drops of oil of cassia in one c. c., this agent is effective as an antiseptic in 1 to 2233 parts; that is to say, one whole drop of the oil of cassia would prevent development of bacteria in 2233 drops of infected broth. This explanation will hold good in connection with each agent we have used. Oil of cassia is undoubtedly the most potent of the essential oils as an antiseptic. I have had at least a dozen samples of cassia, obtained from as many different sources, and upon analyzing them have found them to be adulterated in each instance. One sample, especially, shipped direct from China to a dealer in Chicago, was found to contain fixed oils in considerable quantity. Others were found to contain alcohol, etc. This oil, as found in commerce to-day, is not as potent an antiseptic, by about one-half, as was the cassia obtained ten years ago. Reference to the work of Dr. G. V. Black along this same line, done about ten years ago, serves to prove the correctness of this statement. The samples of cassia he used at that time were potent in 1 to 4000 parts. If I could have obtained a pure, unadulterated sample of cassia, it would have certainly outclassed oil of cinnamon as an antiseptic by a wide margin, but as it is, as to the division of a drop, they have proven exactly the same. However, you will notice when we consider that agent, that of the oil of cinnamon, only sixty-three drops are required for one c. c., while of cassia sixty-seven drops are required. This simply means that one drop of the oil of cinnamon is just slightly larger in bulk than one drop of the oil of cassia, so that this discrimination in the number of drops to the c. c. still places the oil of cassia ahead of the oil of cinnamon as an antiseptic, the potency of oil of cinnamon figuring out 1 to 2100 parts. While oil of cassia heads the list of the essential oils as an antiseptic, it is also true that it is the most poisonous in its effect upon soft tissue. As a test of the irritating properties of oil of cassia, a pellet of cotton was saturated with it and placed in a small rubber cup to prevent evaporation. This was applied to the surface of the skin and held there by means of a piece of court plaster large enough to cover it over and stick tightly to the surface of the skin about the edges. This was retained in place for twenty-four hours, during which time the irritation to the soft parts was by no means a pleasant feature. At the end of this period a blister invariably forms, however, the inflammation in the tissues at this time is not very great. The blister will occupy an area from one-half to one-third greater than that to which the oil is directly applied, and will fill and refill with serum several times before any tendency to recovery is noticed. At the end of forty-eight hours the inflammation in the parts involved is intense, and occupies an area four or five times as great as that to which the oil is directly applied. Numerous small independent blisters almost invariably form about the circumference of the inflamed area. This condition continues for several days, and while the inflammatory process is at its height the sore is one of the ugliest and most formidable in appearance it has ever been my privilege to look upon. These sores, also, are very slow in healing. It is with seeming regret on their part that the inflammation is permitted to subside, and the parts to return to a normal condition. While these sores are in every way just as bad as has been described, they are, however, fraught with no serious consequences.

To further test the irritating properties of this oil, a sore, in connection with which there was considerable inflammation, was produced on a guinea pig and treated for a number of days with the spray of cassia by means of an atomizer. So long as this treatment was continued the parts could not recover, but quite to the contrary, the inflammation was greatly increased. Suppuration was then produced by infecting the sore with pus microbes. This in turn was treated with the spray of cassia, with the result that the germs were destroyed, and the pus formation ceased, thus proving quite conclusively that this agent is an excellent germicide, when applied to suppurating surfaces, as well as a most potent antiseptic.

To my mind it is clearly proven that while the antiseptic and germicidal properties of this oil are of the highest order, it is one of the most irritating in its effects on soft tissue of all the agents with which we have anything to do. And I feel perfectly justified in making the statement that oil of cassia should never be used as a dressing in the root-canals of teeth.

There is another reason aside from the above why it should not be used, and that is, its proneness to cause discoloration of the teeth. In almost every instance in which its use is continued for a time the teeth are more or less discolored, and in some cases very considerably. This is one of the most difficult forms of discoloration to correct that we are called upon to treat.

Is it not reasonable to suppose that when cassia is used in the treatment of pulpless teeth that the above disagreeable conditions may occur in the soft tissues occupying the apical space, and the peridental membrane become involved in the inflammatory process? Have you ever thought that the excessive flow of serum, which so frequently occurs from the tissues of the apical space of teeth that are being treated with this oil, is nothing more or less than the discharge of actual blister as in the cases above recited? If these are reasonable suppositions—and I believe they are—is it still a source of wonder to any of you that teeth, under these circumstances, so suddenly develop such extreme tenderness to pressure as they so frequently do?

Oil of cassia, however, has a place in our practice as dentists. Cassia water, sometimes, in the treatment of fistulous abscesses is very useful. It is so stimulating to the tissues that it excites a healthy action on the part of the latter when other agents fail. Oil of cassia in the treatment of severe cases of pyorrhea, so-called, where the pockets about the teeth are deep and considerable pus present, is exceedingly useful. In such cases it may be used in full strength by means of a drop-syringe. The oil is not permitted to remain in contact with the soft tissues a sufficient length of time to cause trouble, it is so soon diluted by the fluids of the mouth.

Oil of Cinnamon of Ceylon.—We find that $\frac{3}{10}$ of a drop prevents development of bacteria in the *unit of culture media*, and that sixty-three drops constitute one c. c., thus showing this agent effective as an antiseptic in 1 to 2100 parts. Oil of cinnamon of Ceylon, as you well know, is very much the same nature as oil of cassia. However, in some respects there is a marked difference between them. It has been demonstrated that oil of cinnamon is not so irritating to soft tissue as oil of cassia. An application to soft tissue in the same manner that cassia was applied, and left for twenty-four hours, caused considerable irritation and formation of blister. At the end of forty-eight hours the inflammation was severe; however, not so intense as that caused by cassia, and the area of tissue involved in the inflammatory process was not so great. Also, the blister that developed by the application of cinnamon was by no means as large as that from cassia, occupying the center of the inflamed area and spreading over tissue in extent equal only to that to which the agent was directly applied. The blister and inflammation are not so persistent as is the case with cassia, the former refilling with serum usually but two or three times, and the inflammation passing away quite readily.

A sore, attended with much inflammation, on a guinea pig, was treated with the spray of oil of cinnamon with the result that it was further constantly irritated and thus prevented from healing. Suppuration was then produced in the sore and again treated with the spray of this oil, the germs being destroyed and the pus formation ceasing. The action of cinnamon was not so vigorous as that of cassia.

To my mind, cinnamon is altogether too irritating for use in the treatment of pulpless teeth.

A Synthetic Oil of Cinnamon, a sample of which I secured this spring from the first lot sent to this country (it being prepared in both France and Switzerland), proves to be as potent an antiseptic as the regular oil, $\frac{3}{10}$ of a drop preventing development of bacteria in the *unit of culture media.* Sixty-four drops of this oil constitute a c. c., thus showing it effective as an antiseptic in 1 to 2133 parts. It is, however, in its first effects more irritating to soft tissue than the oil of cassia. An application was made to soft tissue and at the end of fifteen hours a fully developed blister, in extent larger than the area of tissue to which the oil was applied was the result. There was very little inflammation or discoloration of the tissues. The first effect of this oil on the soft tissue was so vigorous, very much tenderness and inflammation were confidently expected to follow. In this, however, I was disappointed. The blister continued to refill with serum several times, but actually no tenderness or inflammation worthy of mention developed in the surrounding parts. I cannot recommend it for use in the treatment of pulpless teeth.

Beech-Wood Creasote is the next agent, from point of potency, as an antiseptic; $\frac{5}{10}$ of a drop prevented development of bacteria in the *unit of culture media.* There are sixty-four drops in one c. c., thus showing creasote effective as an antiseptic in 1 to 1280 parts. This agent is practically non-irritating to soft tissue. An application remaining for a period of thirty-six hours produced practically no irritation. There was just the slightest evidence of irritation about the center of the spot where it was applied. There was no inflammation. The surface of the skin was slightly discolored and also slightly burned or seared over, but not to an extent that caused the loss of any tissue. A sore on a guinea pig was treated with the spray of creasote with the result that the inflammation gradually subsided, and the sore healed with little delay.

Another sore in which suppuration was produced was treated in like manner, the germs being readily destroyed and the pus formation stopped. Continued treatment resulted in the gradual healing of the sore. Creasote has proven its right to stand among the first, from point of potency, as an antiseptic, and because it has been demonstrated that it is practically non-irritating to soft tissue it is a safe agent, and in some cases a very desirable one, for use in the treatment of pulpless teeth. A case of putrescent pulp, for instance, of long standing, one in which the lateral openings and also the dentinal tubuli are completely saturated with mephitic odors and gases, creasote, in my judgment, is the most effective and desirable of the available agents. It is very penetrating and one of the most persistent in its effects of all the agents at our command. I have used it to good advantage in severe cases of apical pericementitis. However, in some instances, where discoloration of the teeth has occurred, it has seemed that it was due to the action of the creasote. Creasote, being more or less of the nature of carbolic acid, possesses to a certain extent the properties of a local anaesthetic, and because of this property it has quite a beneficial effect upon inflamed tissue.

Oil of Cloves.—$\frac{6}{10}$ of a drop prevented growth in the *unit of culture media;* sixty-nine drops constitute one c. c., showing it effective as an antiseptic in 1 to 1150 parts. Oil of cloves is absolutely non-irritating to soft tissue. An application to the surface of the skin for thirty-six hours left no more evidence of having been confined there than so much sterilized water would have done. No irritation, no discoloration. Sores were produced on guinea pigs and treated with the spray of oil of cloves. The inflammation subsided more rapidly than when treated with any other agent, and the sores healed as rapidly. A sore in which suppuration was produced by being infected with pus microbes was treated with the spray of this oil; the germs were destroyed and the formation of pus was stopped, simply proving beyond any possibility of doubt that while effectively destroying microbes, the only action of the oil in contact with irritated, inflamed soft tissue is that of a quieting, soothing agent, serving to reduce the irritation and inflammation and returning the disturbed tissue to its normal condition.

A sore on my arm, produced by an application of cassia, became infected and pus formed. This was washed thoroughly with a 1 to 1000 solution of bichloride of mercury every night for several times, and dressed in turn with iodoform, nosophen and aristol, with no other result than an absolute failure to stop pus formation. One night, after having washed the sore thoroughly with the bichloride solution, I poured oil of cloves into the raw tissue. There was only a very slight smarting for a few minutes, after which its action was that of a quieting, soothing agent. The application was held in position for twenty-four hours. It was then removed; no pus was present and the little granulations could be seen springing up all over the surface of the sore. It was immediately dressed with aristol and let alone for forty-eight hours, at the end of which time it was perfectly healed.

Another sore on the lower part of my right leg, the result of an application of formalin, was causing a great deal of trouble. The inflammation was severe, the tissues were very sore, the muscles felt bound up and were painful, it being exceedingly difficult to walk. Continued treatment with ordinary remedies resulted in no relief. One morning, after having thoroughly cleansed the sore, a liberal quantity of oil of cloves was placed on it and the bandage applied. Within four hours' time the very disagreeable, drawn condition of the muscles passed away, the pain ceased, and the foot could be moved in all directions as freely and comfortably as could the other, and could be used in walking just as well as it ever could.

Oil of cloves, for general use in the treatment of pulpless teeth, is certainly one of the best agents at our command. It possesses the property of destroying or rendering inert septic and infectious material. In cases of apical pericementitis it is perhaps the best agent that can be used. It possesses local anaesthetic properties to a marked degree, and, like some of the other agents, because of this fact serves to reduce the inflammation in the tissues in the apical space and causes them to return to a normal, healthy condition.

Oil of Bay.—$\frac{7}{10}$ of a drop prevented development in the *unit of culture media.* Seventy-two drops are necessary for one c. c., showing this agent effective as an antiseptic in 1 to 1028 parts. Oil of bay, to me, is a comparatively new agent, and I believe I am warranted in making the statement that it is a new agent to the vast majority of the dental profession. A year ago last winter a gentleman spoke to me about oil of bay, said he had been using it for some time in the treatment of pulpless teeth, and that so far as his clinical experience went had found it to be an agreeable and efficient agent. He stated that he had not observed any bad effects along the line of producing irritation, or anything of that sort. He requested that I test it, which I did, with the result above stated, which places this oil in the foremost ranks of the list of antiseptics. I have used it more or less since, and in one case that I have in mind, thought the irritation and tenderness which was induced was directly due to the action of the oil. But in subsequent use have observed none of these bad effects. I came to the conclusion that I was wrong, that there must have been some foreign, irritating substance present which caused the trouble. I have made two applications of the oil to soft tissue, retaining each in contact for thirty-six hours, for the purpose of observing its effect, and no irritation resulted in either case.

A sore was produced on a guinea pig with an irritant which caused intense inflammation. This was treated with the spray of bay for several days, and the closest observation did not reveal any additional irritation, but to the contrary the inflammation gradually subsided. However, not so rapidly or willingly as when some other agents were used—as cloves. A sore in which suppuration was produced, on being treated with the spray of bay, yielded very nicely, the germs being destroyed and the pus formation stopped. I think we are safe in concluding that oil of bay is a valuable addition to our list of agents for the treatment of pulpless teeth. So far I can see there is no objection to its use, and it is certainly a most effective agent.

Oil of Sassafras.—$\frac{7}{10}$ of a drop prevented development of bacteria in the *unit of culture media.* Seventy drops are required for one c. c., showing it effective as an antiseptic in 1 to 1000 parts. Oil of sassafras in contact with soft tissue for thirty-six hours produced no evidence of irritation. It has proven to be a very potent antiseptic. I have treated sores in which there was marked inflammation with the spray of sassafras, and the result was much the same as with the last previous agents; the inflammation subsiding, the irritation passing away and the sore healing. It has not exhibited the ability to destroy germs and prevent pus formation to nearly the extent that the stronger agents have. I have never used oil of sassafras in the treatment of pulpless teeth, but I certainly can see no reason why it should not be a potent and harmless agent in this connection.

OIL OF PEPPERMINT.—$\frac{8}{10}$ of a drop prevented development of bacteria in the *unit of culture media.* Seventy-two drops are necessary for one c. c., showing this agent effective as an antiseptic in 1 to 875 parts. An application of oil of peppermint to soft tissue continued for thirty-six hours produced no irritation, no discoloration of the skin, no inflammation, thus showing conclusively that this, also, is non-irritating to soft tissue. A sore in which considerable inflammation was present was treated with the spray of this oil, with the result that the inflammation readily yielded, the irritation subsided and the sore healed. Another sore in which suppuration was produced was treated in the same way, with the result that the germs were destroyed and the pus formation stopped, which proves that this agent is not only an antiseptic, but also destroys the germs and thus prevents pus formation. This is an agent which I have rarely ever used in practice. Three years ago I used it a little in treatment cases, but discarded it simply because of its persistent, penetrating odor. Other than that I can see no objection to its use in pulpless teeth.

DR. BLACK'S I–II–III.—This is the next agent in point of potency. $1\frac{4}{10}$ drops prevented development in the *unit of culture media.* Sixty-five drops are necessary for one c. c., showing this agent effective as an antiseptic in 1 to 454 parts. I–II–III, as you well know, is a preparation given to the profession a number of years ago by Dr. G. V. Black, consisting—the mild solution, so-called, and this is the one used in these tests—of one part oil of cassia, two parts carbolic acid crystals and three parts gaultheria. It has always proven itself a most efficient agent in the treatment of pulpless teeth, and has been used by very many in the dental profession for the last ten or twelve years, possibly, more than any other one agent. I have used it continuously since I have been in practice, and have never observed any bad effects from its use. No irritation of the soft parts, no tenderness of the tooth to pressure, no inflammation resulting. Possibly some of you will wonder why I–II–III is such an efficient and desirable agent, consisting, as it does, of cassia, carbolic acid and gaultheria; carbolic acid being not a positive persistent antiseptic, but one whose restraining effects upon the development of bacteria are only transient; oil of gaultheria being absolutely worthless as an antiseptic, and the use of cassia being so thoroughly condemned because of its extreme irritating properties. Of course this agent depends upon the cassia for its antiseptic properties. The gaultheria is used as a diluent to the cassia. The carbolic acid was used more especially because of its anaesthetic properties on soft tissue. When these different agents are properly mixed to form I–II–III, it is the opinion of Dr. Black that there is more or less of a chemical union between them, so that the individuality of each separate agent seems to be lost, and the result is the formation of a new agent, or one with different characteristics from those possessed by the three individual agents. At any rate, it is non-irritating to soft tissue. An application left on for thirty-six hours produced no irritation whatever. There was only a slight searing and discoloration of the surface of the skin. Sores with much inflammation present were treated with the spray of I–II–III, which did not produce further irritation. Its action was more like that of a neutral agent (so to speak), not irritating the sore, nor, on the other hand, imparting, to any appreciable extent, a soothing, quieting influence, the inflammation subsiding just about as it would if left to itself with all irritating influences removed. A sore in which suppuration was produced was treated with the spray of this agent. It demonstrated its right to be classed as a very potent germicide. The germs were destroyed and the pus formation ceased.

I–II–III, as formed with the present cassia of commerce, is not so potent an antiseptic as that formed with cassia obtainable several years ago. This must be due to the fact above stated that cassia is so adulterated at the present time. In fact, I–II–III, is lessened in potency in almost direct proportion to the extent of the adulteration of the cassia.

Seven-tenths of a drop was effective in ten c. c. of broth, as shown by experiments conducted by Dr. Black several years ago.

I–II–III, as shown by these experiments, is abundantly effective, but if cassia is continued to be adulterated the time may come when it will not be. For general use, in the treatment of pulpless teeth, I–II–III is certainly an effective and excellent agent.

Carbolic Acid, 95 Percent.—$1\frac{8}{10}$ drops prevented development in the *unit of culture media.* Sixty-one drops are required for one c. c., showing this agent effective as an antiseptic in 1 to 338 parts. Carbolic acid is not a permanent, positive antiseptic. Its restraining power on the development of bacteria, in the majority of plants one makes, is only transient. $1\frac{8}{10}$ drops prevented development for a period of three days, after which the bacteria developed in almost every instance. The restraining effect of this agent upon the development of bacteria seems to be almost in direct proportion to the quantity of the agent used in the culture tube.

Oil of Myrtol.—$1\frac{9}{10}$ drops were necessary to prevent development of bacteria in the *unit of culture media.* Sixty-eight drops constitute one c. c., showing myrtol effective as an antiseptic in 1 to 357 parts. Oil of myrtol is an agent which I have used but little in practice. In the majority of cases in which I have used it, there has been more or less irritation produced, more or less tenderness of the tooth developing, so that it impressed me as being somewhat of an unfavorable agent for this purpose. An application of myrtol to soft tissue for thirty-six hours produced decided irritation, and there was a strong tendency to the formation of blister. The surface of the skin was destroyed. The irritation and inflammation present continued for two or three days, gradually abating. A sore on a guinea pig being treated with the spray of this oil showed evidence of further irritation. So long as the treatment was continued the inflammation refused to subside. A suppurating sore, being treated in the same way, was certainly benefited by a consequent destruction of the germs and cessation of pus formation. There is no doubt but that this agent is quite irritating, and one that should not be generally used in the treatment of pulpless teeth. There are cases in which I use strong myrtol water, seemingly to good advantage, and these are in connection with abscesses with fistulous openings, especially those of long standing, in which there is more or less irritation of the soft parts throughout the tract of the fistule, and that uneasy, disagreeable sensation often experienced by the patient in connection with these cases.

Oil of Cajuput.—6 drops are necessary to prevent development in the *unit of culture media.* Seventy-two drops are necessary for one c. c., showing this agent effective as an antiseptic in 1 to 120 parts. Cajuput is non-irritating to soft tissue. Applications of this oil to soft tissue, retained for thirty-six hours, produced no evidence of irritation, in fact, the discoloration of the skin was very slight and remained but a short time. A sore on a guinea pig, in which there was considerable inflammation, was treated with the spray of this oil, and no increase of the irritation was produced. Another sore in which suppuration was produced was treated in the same way, with the result that the germs were gradually destroyed, its action, however, not being positive, for if the treatment was discontinued for a day or two the pus formation continued as before.

Oil of cajuput is an agent which I have never used very extensively in my practice. At first I did use it more or less in the treatment of pulpless teeth, but latterly I have not used it in this connection; in fact, the only use I make of it is occasionally to moisten the inner walls of the root-canals previous to filling with gutta-percha. For this purpose its non-irritating nature recommends it, and especially the fact that it is a solvent of gutta-percha and causes the latter to adhere to the walls of the canals.

Eucalyptol (Sander's and Merck's).—6 drops each of these preparations are necessary to prevent development in the *unit of culture media.* Seventy drops are necessary for one c. c., showing each preparation effective as an antiseptic in 1 to 116 parts. Eucalyptol in contact with the skin for thirty-six hours produced no evidence of irritation, no inflammation, no discoloration, thus proving that the agent is non-irritating and harmless in contact with soft tissue. A sore in which considerable inflammation was present was treated with the spray of this agent, with the result that the inflammation readily yielded, the irritation subsided and the sore healed, thus further proving that it is non-irritating even to injured, inflamed soft tissue. A sore in which suppuration was produced was treated in the same way, with virtually the same results as with cajuput; it exhibited a restraining influence upon the development of bacteria and pus formation, but the treatment being discontinued for a while pus formation went on as before. As an agent to place in the root-canals of teeth after the removal of a pulp, following devitalization, in order to keep the parts healthy for a few days previous to root-canal filling, it is perhaps the agent that I use more than any other. It is certainly harmless, never exciting irritation. For the purpose of slightly moistening the inner walls of root-canals previous to filling, eucalyptol is the agent that I nearly always use.

Oil of Eucalyptus as found in the market only produced a restraining effect upon the development of bacteria when a saturated solution was formed with the bouillon.

Oil of Gaultheria was carried in my experiments as high as 8 drops, this quantity forming a saturated solution in the *unit of culture media*, that is to say, the broth had taken up, or dissolved, all of the oil that it could possibly retain, there being also a large number of free globules floating about in the broth, and still development of bacteria took place quite abundantly, showing that this agent is useless in restraining the development of bacteria. It is certainly of no use to us as an antiseptic.

Eugenol.—This agent resulted in the same way as gaultheria. Eight drops were used in the *unit of culture media*, which amount formed a saturated solution with numbers of globules of the free oil floating about. and still the bacteria developed, thus proving that eugenol, also, is useless an an antiseptic.

FORMALIN.—Of late the dental profession has taken up this agent for the treatment of pulpless teeth, the treatment of abscesses, and for devitalizing pulps, etc., and many are reporting wonderful results from its use. Not long since I read an article in one of our journals in which the writer paid a glowing tribute to this agent as a most efficient and desirable one for the treatment of almost all conditions of pulpless teeth. Having had some experience with it myself, and because of many negative results experienced, having had my suspicions aroused as to whether it was a proper agent to be used about the mouth, I decided to investigate it as thoroughly as possible. First I tested it as to its antiseptic properties, and found it to be quite a powerful antiseptic. Of the formalin preparation, which is a saturated solution of the gas formaldehyde in water (the latter taking up about 40 percent of the former), $\frac{4}{10}$ of a drop prevented development of bacteria in the *unit of culture media.* Fifty-six drops are necessary for one c. c. This shows formalin potent as an antiseptic in 1 to 1400 parts. Somebody has been so enthusiastic over this agent as to make the statement that it is fully as potent an antiseptic as bichloride of mercury. This is certainly a mistake. I prepared a 1 to 1000 solution of bichloride of mercury and found that it required nine drops of this solution to prevent development of bacteria in the *unit of culture media.* I prepared a 1 to 1000 solution of pure formaldehyde, which we now have in a solid state—the gas being reduced to such by chemical processes—and of this solution found that it required 40 drops to prevent development of bacteria in the *unit of culture media,* thus proving that formaldehyde is not so potent an antiseptic as bichloride of mercury, by at least four-fold. I next resolved to determine its ability to irritate soft animal tissue—the same as I did with the other agents. I took a small pellet of cotton, saturated it with formalin, placed it in a small rubber cup to prevent evaporation, placed it on the surface of the skin on the lower part of my right leg, and covered it over with a large piece of court plaster stuck tightly about the edges. This was placed there the 14th day of last March at 12:30 a.m. I went to bed and went to sleep. Between four and five in the morning I was awakened by the pain, and could get no rest afier that. The pain was quite intense and of a very peculiar character. It seemed as if something were inside my leg gripping it as if in a vise. Then it would take a turn and twist about, as if tearing the inside out. It would stop for an instant, and then the performance would be repeated with renewed vigor. The pain continued more or less severe all day. I wanted to keep the application in place for twenty-four hours—the time adopted for the other agents—but at the end of twenty hours, the pain had been so constant and the tissues began to look so ugly, that I concluded to remove it. The tissue to which it was applied and for about two inches in all directions was turned as white as pure snow, as if all the blood were driven from the parts. The pain was lessened very considerably within a short time after the application was removed. The tissue to which it was directly applied was perfectly anaesthetized to a considerable depth. Just at the circumference of the application there was considerable tenderness. There was much swelling which seemed to be more like that of œdema than of true inflammation. In about two or three days some color began to return to the parts, except to which the agent was directly applied, which latter never regained normal color. In about two days more a line, purple in color, began forming at the circumference of the point of application—a line of demarcation—and it became apparent that there was to be a break in the tissues. This break occurred and sloughing took place; considerable tissue was lost all over the surface of the inflamed area. The tissue in the center raised about the edges, but was very obstinate about coming away. From the time the agent was thoroughly absorbed in the tissues, physically I was not up to the standard; my appetite was more or less impaired, the digestive and eliminative organs were somewhat interfered with. These conditions continued to grow worse until the climax came in quite a severe case of systemic poisoning, the poisonous matter being thrown off through the medium of quite a severe diarrhoea, and also much vomiting—the former continuing for a period of three days, the latter for one day, following which time my physical condition rapidly improved.

Having seen a number of cases which have been treated by physicians with various percent solutions of formalin in which more or less sloughing of the parts has resulted—one which I saw not long since in which as low as a 2 percent solution was used, in connection with which considerable sloughing resulted—and also because of the very vivid recollections of my own personal experiences with it, I have come to the conclusion that we should get along without it in the treatment of diseased conditions about the mouth.

